# PD1-Expressing T Cell Subsets Modify the Rejection Risk in Renal Transplant Patients

**DOI:** 10.3389/fimmu.2016.00126

**Published:** 2016-04-11

**Authors:** Rebecca Pike, Niclas Thomas, Sarita Workman, Lyn Ambrose, David Guzman, Shivajanani Sivakumaran, Margaret Johnson, Douglas Thorburn, Mark Harber, Benny Chain, Hans J. Stauss

**Affiliations:** ^1^Institute of Immunity and Transplantation, University College London, London, UK; ^2^Department of HIV Medicine, Royal Free London NHS Foundation Trust, London, UK; ^3^Sheila Sherlock Liver Centre, Royal Free London NHS Foundation Trust, London, UK; ^4^Department of Renal Medicine, Royal Free London NHS Foundation Trust, London, UK

**Keywords:** transplantation, rejection, T cells, protein death 1, risk factor

## Abstract

We tested whether multi-parameter immune phenotyping before or after renal ­transplantation can predict the risk of rejection episodes. Blood samples collected before and weekly for 3 months after transplantation were analyzed by multi-parameter flow cytometry to define 52 T cell and 13 innate lymphocyte subsets in each sample, producing more than 11,000 data points that defined the immune status of the 28 patients included in this study. Principle component analysis suggested that the patients with histologically confirmed rejection episodes segregated from those without rejection. Protein death 1 (PD-1)-expressing subpopulations of regulatory and conventional T cells had the greatest influence on the principal component segregation. We constructed a statistical tool to predict rejection using a support vector machine algorithm. The algorithm correctly identified 7 out of 9 patients with rejection, and 14 out of 17 patients without rejection. The immune profile before transplantation was most accurate in determining the risk of rejection, while changes of immune parameters after transplantation were less accurate in discriminating rejection from non-rejection. The data indicate that pretransplant immune subset analysis has the potential to identify patients at risk of developing rejection episodes, and suggests that the proportion of PD1-expressing T cell subsets may be a key indicator of rejection risk.

## Introduction

Transplantation remains a life saving treatment for patients with kidney failure. Due to improvement in organ preservation, advances in surgical technologies, and the use of potent immune suppressive treatment regimens, the incidence of acute rejection has dramatically decreased in the recent past. However, despite potent immune suppression, approximately 20% of patients still develop acute rejection episodes (1). Such episodes predispose to chronic antibody-mediated rejection, tubule-interstitial fibrosis, and atrophy, resulting in irreversible damage and failure of the transplanted kidney. A registry study of 63,045 renal transplant patients showed that acute rejection episodes were the single most important predictor of chronic allograft nephropathy, increasing the risk of graft failure by 5.2-fold compared to patients without episodes of acute rejection (2). The loss or damage of a renal transplant secondary to rejection has substantial consequences for patients in terms of increased all cause mortality. Acute rejection episodes result in an increased burden of immunosuppression contributing to the high rate of infectious and cancer-related deaths in the transplant population. Furthermore, graft loss has very substantial financial repercussions, as the annual cost of providing dialysis is approximately six times that of supporting a stable transplant.

Because of the importance of avoiding rejection and the consequences of unnecessary over-immunosuppression there is an ongoing search for biomarkers, which can identify the risk of rejection. Preexisting antibodies to donor HLA are an established risk factor for acute rejection (3). Soluble CD30, a member of the TNF receptor superfamily, which is expressed on T cells and shed into blood, has received considerable attention, but the predictive power of this marker is still not clear (4). More recently, a transcriptomic analysis has identified a panel of 17 genes whose expression can identify rejection episodes without the need for biopsy, and can predict rejection episodes up to 3 months before rejection can be observed histologically (5). The screening for expression of a set of five genes has been similarly used to identify patients with episodes of acute rejection (6). We were particularly interested in exploring whether pre-transplantation immune-profiling might provide a tool to predict those patients at risk of rejection, which would inform patient management and allow clinicians to adjust the dosing parameters of immunosuppressive medication accordingly.

Hence, we have performed a multi-parameter flow cytometry analysis of adaptive and innate lymphocyte subsets in the peripheral blood of renal transplant patients before and in the first 12 weeks after transplantation. Using statistical machine learning algorithms to analyze this complex data set, we find that the pretransplant immune-phenotype predicts the risk of acute rejection episodes, and that the proportion of PD1-expressing regulatory and conventional T cells is a key component of the predictive signature. These results suggest a strategy for developing personalized immune suppressive regimes according to the predicted rejection risk assessed prior to transplantation.

## Materials and Methods

### Patients

The Immune Monitoring Study was conducted at the Royal Free Hospital, London, between May 2011 and October 2014. The study protocol was approved by the National Research Ethics Committee. All patients (*n* = 28) had given written informed consent to participate in the study, and participants were of diverse age and ethnicity. Clinical details and patient demographics are shown in Table 1. Blood samples were collected from patients pretransplant and posttransplant. For live donor organ recipients, pretransplant samples were taken before immunosuppression was started, and on the day of transplant. For cadaveric donor organ recipients, pretransplant samples were taken before immunosuppression on the day of transplant. After transplant, weekly samples were taken until week 12 posttransplant for all patients. All samples were taken before starting immunosuppression.

**Table 1:** **Further clinical details and demographic of patients included in this study**.

No.	Donor	Age	Ethnicity	CMV status	HLA ­mis-match	Original disease	Dialysis or not	Sensitization	Post-tx CMV viremia	Rejection	Other information
1	Live	33	White	D+ R−	111		Dialysis	Donor specific Abs: low level DQ2	Yes	Yes, cellular, 6 months	

2	Cadaveric	51	White	D+ R+	121	ADPKD	Dialysis	Not sensitized	Yes, D7	Yes, cellular, W1–2	

3	Live	49	White	D− R−	112	Familial Hyperuricaemic nephropathy	Predialysis	Not sensitized	No	Yes, cellular, W9	

4	Cadaveric	72	Black African	D+ R+	121	Small kidneys	Dialysis	Not sensitized	Yes, W4–6	Yes, cellular, W2	

5	Cadaveric	49	White	D− R+	110	MPGN	Dialysis	Not sensitized	No	Yes, cellular, W2 and W18	

6	Live	63	White	D+ R+	000	Small kidneys	Dialysis	Not sensitized	No	Yes, cellular, 12 months	

7	Cadaveric	25	Black African	D+ R+	111	Reduced nephron mass and hypertension	Dialysis	Not sensitized	No	Yes, cellular, 2 years	ABOi, baseline postimmunosuppression

8	Live	52	White	D+ R+	122	ADPKD	Predialysis	Sensitized B8, B16, B35, A33	No	Yes, cellular, 2 months	
								No DSA			

9	Cadaveric	40	White	D+ R+	022	Proliferative glomerulonephritis	Dialysis	Not sensitized	Yes, low	Yes, cellular, W2	

10	Live	46	White and Black Car.	D+ R−	121	AA amyloid	Predialysis	Not sensitized	No	Yes, Ab-mediated, W1–3	HCV^+^

11	Live	50	Black African	D+ R+	011	Type-2 diabetes	Dialysis	Not sensitized	Yes	Yes, cellular, W1	

12	Live	39	White	D+ R−	011	ADPKD	Predialysis	DSA low level DR1	Yes	No	

13	Live	33	White	D+ R+	111	Small kidneys	Predialysis	Not sensitized	No	No	HBV^+^

14	Cadaveric	57	White	D+ R+	211	Small kidneys	Dialysis	Not sensitized	Yes, W7–9	No	

15	Live	33	Black African	D+ R+	111	Small kidneys	Dialysis	Sensitized cw5, cw7, B8. No DSA	No	No	

16	Live	65	Black	D− R+	011	Ischemic nephropathy	Dialysis	Sensitized B82, B81, B55, B54, B42. No DSA	Yes	No	

17	Live	26	White	D− R−	111	HSP	Predialysis	Not sensitized	No	No	

18	Live	46	Asian	D+ R+	222	IgA nephropathy	Predialysis	Not sensitized	Yes, W10–12	No	

19	Live	31	Asian	D− R−	110	Small kidneys	Predialysis	Sensitized A34	No	No	
								No DSA			

20	Cadaveric	53	Asian Indian	D+ R+	111	Renovascular disease	Dialysis	Not sensitized	No	No	

21	Live	38	Mixed	D− R+	210	IgA nephropathy	Predialysis	Not sensitized	No	No	

22	Live	23	White	D+ R−	000	Renal dysplasia	Dialysis	Sensitized B45, B76, B82, B44, A1	Yes, W7–9	No	
								No DSA			

23	Cadaveric	40	Black African	D− R+	220	Hypertension	Predialysis	Sensitized B52, B45	No	No	
								No DSA			

24	Live	33	White British	D− R−	111	Type 1 diabetes	Dialysis	Not sensitized	No	No	

25	Live	46	Any other white British	D+ R+	111	HSP	Dialysis	Sensitized A11	No	No	
								No DSA			

26	Live	23	Black African	D+ R+	000	Vasculitis	Predialysis	Not sensitized	No	No	

27	Live	46	Any other black	D+ R+	001	Hypertension	Predialysis	Not sensitized	No	No	

28	Live	38	Other – not stated	D+ R+	110	IgA nephropathy	Predialysis	Not sensitized	No	No	

*W, week; D, day; ABOi, ABO incompatible; HCV, hepatitis C virus; HBV, hepatitis B virus; HIV, human immunodeficiency virus; Car., Caribbean; DSA, donor-specific antibody; ADPKD, autosomal dominant polycystic kidney disease; HSP, Henloch–Scholein Purpura; MPGN, membranoproliferative glomerulonephritis; CMV, *Cytomegalovirus*. Patients 1–23 were used as the initial test cohort, and patients 23–28 were included in the validation cohort*.

Patients received the following immunosuppressive medications: 20 mg Basiliximab monoclonal antibody (anti-CD25) therapy on the day of transplant and on day 4 after transplant. Five hundred milligram intravenous methylprednisolone on the day of transplant followed by 40 mg intravenous methylprednisolone for 3 days after transplant, then 20 mg of oral prednisolone for 7 days, followed by 7 days of 5 mg oral prednisolone. Tacrolimus was given with dose adjusted according to plasma levels. 1 g of Mycophenolate Mofetil for 1 month, 750 mg for a further 2 months, reduced to 500 g by 3 months posttransplant. Live donor recipients received both Tacrolimus and Mycophenolate Mofetil from ~2 weeks before transplant, while cadaveric donor recipients received these from the day of transplant. Both groups of patients continue with these medications indefinitely.

### Rejection

In cases of unexplained increased serum creatinine levels, patients underwent kidney biopsy, and rejection of the kidney allograft was confirmed by histological analysis. Rejection was either cell-mediated (characterized by infiltration of lymphocytes and inflammatory cells into the organ) or antibody-mediated (characterized by C4d deposition in the organ and circulating donor-specific antibodies). Rejection was treated with steroids and/or modification of maintenance immunosuppression.

### Blood Samples

Peripheral blood mononuclear cells (PBMCs) were separated from whole blood by means of density gradient centrifugation, and were stored at −180°C in vapor-phase nitrogen in the UCL-RFH Biobank. Samples were analyzed for flow cytometry no more than 3 years after storage.

### Flow Cytometry

Multi-parametric flow cytometry was used for immunophenotyping. PBMC (1 × 10^6^) were stained with a T-cell panel, an innate lymphoid panel or with an isotype control panel. Before antibody labeling, cells were incubated with purified human IgG (Sigma) to reduce non-specific binding. Cells were stained with mAbs against CD3-PE Cy7 (clone SK7), CD4-BD Horizon v500 (clone RPA-T4), CD8-BD Horizon v450 (clone RPA-T8), CD45R0-PECF594 (clone UCHL1), CD62L-APC (clone DREG-56), CD25-APC Cy7 (clone M-A251), CD127-FITC (clone HIL-7R-M21), CD279-PE (clone EH12.2H7) (Biolegend), HLA-DR-PerCPCy5.5 (clone LN3) (eBioscience), CD16-APC H7 (clone 3G8), CD56-APC (clone NCAM 16.2), iNKT-PE (6B11), Vδ2-FITC (clone B6), IgG1k-APC Cy7 (clone MOPC-21), IgG1k-APC (clone MOPC-21) (Biolegend), IgG1k-FITC (clone MOPC-21) (Biolegend), IgG1k-PE (clone MOPC-21) (Biolegend), and IgG2b-PerCPCy5.5 (clone N/S) (eBioscience). Antibodies were from BD Biosciences unless stated otherwise.

### Flow Cytometric Analysis

Flow cytometric analysis was carried out using a BD LSRFortessa™ cytometer with BD FACSDiva™ software v.6.0.1 (BD Biosciences). The data were analyzed using FlowJo v7.6.5 software (Treestar Inc.). Gates for CD25^+^, CD127^+^, PD-1^+^, and HLA-DR^+^ CD4^+^ and CD8^+^ T cell populations were set using the isotype control stained samples for each patient to define the negative population.

### Principal Component Analysis

Principal component analysis (PCA) is an exploratory technique that is used to visualize high-dimensional data by projecting the data into a new smaller set of dimensions called principal components (PC), which contain most of the information within the data set. The first dimension of the new data is made up of a linear combination of all the measured dimensions of the data, with the coefficients chosen such as to maximize the variance of the dimension across all the samples. Subsequent PCs contain progressively less of the variance. Each PC is linearly independent uncorrelated to all other PC. Typically most variance is contained within a few PCs, which can be visualized in a series of two-dimensional plots. Since the mapping into the new coordinate system is given by a weighted linear sum of all original input variables (i.e., T cell subset frequencies in peripheral blood), the contribution of each original variable to each PC is reflected by the size of the corresponding weight coefficients.

### Support Vector Machines

Support vector machines (SVM) are supervised binary classification tools (7). Given a set of training data, an SVM seeks an optimal separating hyperplane to split data points from two classes (e.g., rejection vs. no rejection). In order to accommodate non-linear boundaries between the data, a kernel function can be used to transform the original input space into a higher dimension feature space, where linear structure may be found. We initially compared the results of using untransformed data with a radial Gaussian kernel when constructing the SVM. No difference in classification accuracy was observed and all results shown use untransformed data.

The SVM algorithm learns the separating hyperplane such that the distance between the plane and the nearest points from each class, the margin, is maximized, subject to a cost (governed by a tuning parameter C), which penalizes points that falls on the wrong side of the margin. The value of the cost parameter, C, was determined by optimizing model accuracy over a range of values.

The algorithm, which yields the optimal separating hyperplane, is defined by a linear combination of the data dimensions. The linear coefficients defining the hyperplane can be considered as a set of weights, which identify those dimensions of the data with the greatest influence on the classification.

Additionally, the probability of class membership (e.g., ­rejection vs. no rejection) can be calculated by fitting a logistic regression model to the decision values that are output from the SVM (8). The decision values are the Euclidean distances that define how far each patient sample lies from the optimal separating hyperplane. Loosely speaking, the further sample lies from the boundary, the greater the probability that the sample belongs to its predicted class.

### Validation

A key element to evaluate the power of any statistical model in classification is validation. In order to maximize the statistical power from our initial patient sample size (*n* = 23), we evaluated the model using leave-one-out validation. In this approach, one patient is selected, and all results from that patient are removed from the data set. The SVM model is then optimized using the data from the remaining 22 patients, together with their known classification labels (i.e., reject/non-reject). The model is then used to predict the classification of the sample, which had been left out. In this way, the classification algorithm is built without including any knowledge from the patient who is being tested, and this patient serves as an unbiased validation case. The procedure is repeated for each individual patient, and the success rate of the classification is measured over all 23 patient data sets. We also used more traditional train/test strategy. An additional 5 patients were included into our study and independently analyzed by flow cytometry by a scientists who was not involved with the analyses of the initial 23 patients. A repeat flow analysis of a previously studied patient was also included to test reproducibility. The SVM algorithm trained on the initial 23 patients was used to assess the rejection risk of the 6 independently analyzed patients.

All analyzes were performed using statistical programing language R. SVM were implemented using the package e1071, while PCA and hierarchical clustering were performed using the heatmap.2 and prcomp functions in the core library.

Traditional statistical tests for significance were carried out using Mann–Whitney tests with Bonferroni test for multiple testing where required.

## Results

Table 1 shows a summary of the patient cohort included in this study. Of the 28 patients, histologically confirmed rejection was seen in 11 patients and 17 did not show signs of rejection. The CMV status and the percentage of patients with live and cadaveric donor organs were similar in the two subgroups. We did not see an increased incidence of CMV reactivation and viremia in the patients who had rejection episodes. BK infection was not detected in any of the patients.

**Figure 1:**
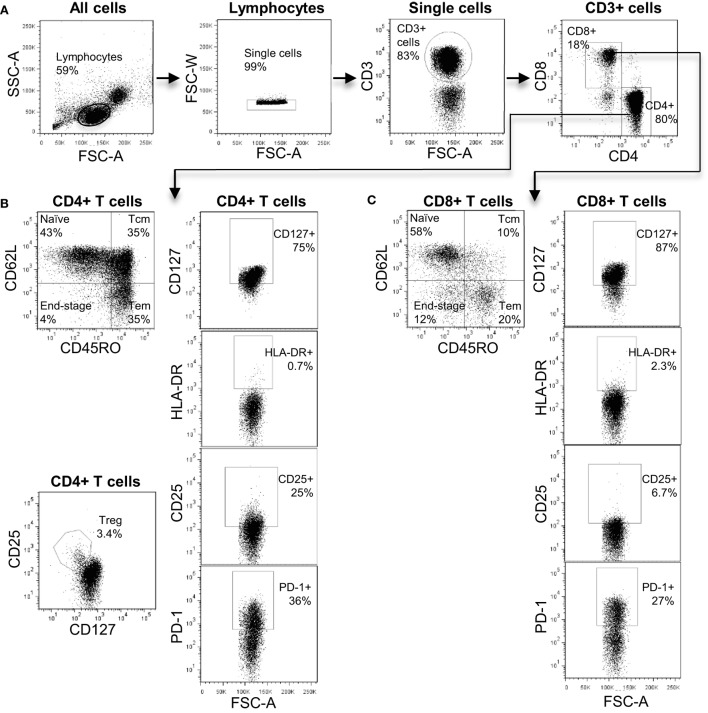
**The T cell immune-phenotyping panel**. **(A)** Flow cytometry plots showing the gating strategy to identify CD4^+^ and CD8^+^ T cell subsets. **(B)** Analysis of CD4^+^ T cell subsets including naive, central memory, effector memory, and end-stage subsets. In addition, the identification of CD4^+^ Treg is shown. **(C)** Analysis of CD8^+^ T cell subsets including naive, central memory, effector memory, and end-stage subsets.

**Figure 2:**
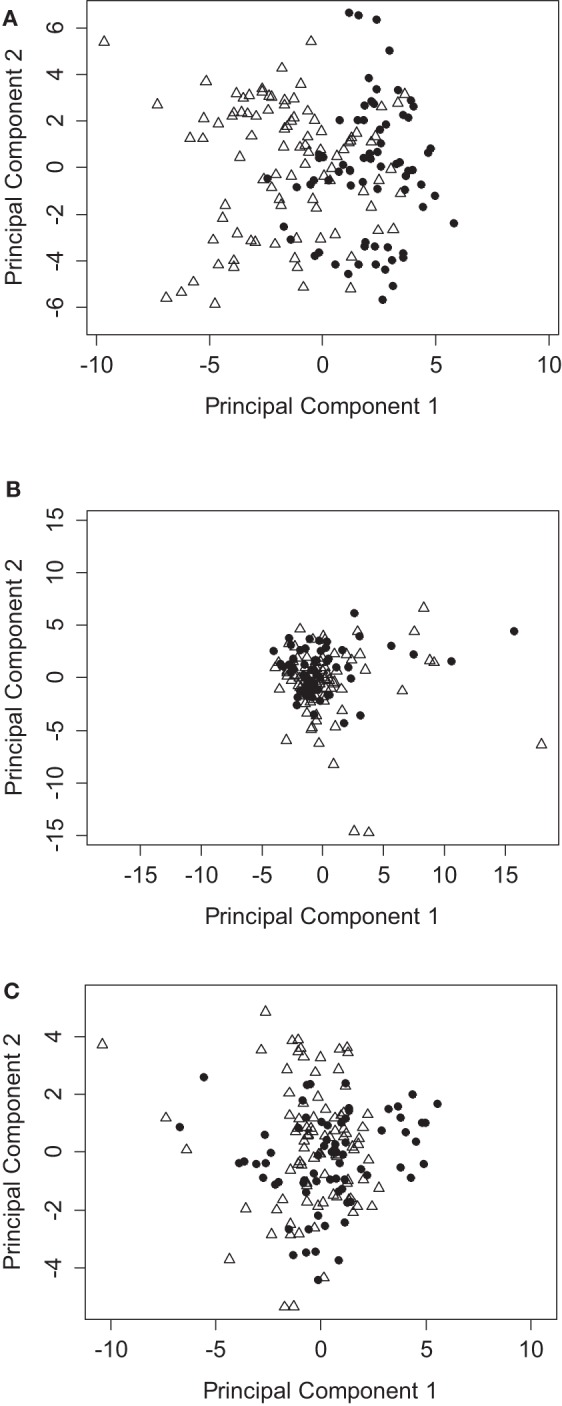
**PCA analysis of immunophenotyping data segregates between patients with or without rejection episodes**. **(A)** PCA using all data points from the T cell immunophenotyping panel, showing the coordinates of samples from patients who reject (triangles) compared to those who do not reject (circles). **(B)** As for **(A)**, but all data points are normalized to pretransplant baseline values. **(C)** As for **(A)**, but using the NK, iNKT, and γ/δ T cell immunophenotyping panel.

The antibodies used in our T cell panel were specific for nine molecules that allowed us to define distinct T cell subsets, and quantify the proportion of activated cells (using CD25 and HLA-DR) and exhausted cells (using PD-1) within each subset. FlowJo analysis of the flow cytometry data was used to extract quantitative data on 52 T cell phenotypes each defined by expression of a particular combination of markers (see Table S1 in Supplementary Material for full list). Figure 1 shows a representative flow cytometry profile obtained after staining with our T cell panel and indicates some of the T cell subsets that were included in the bioinformatics analysis. We used the expression pattern of CD45RO and CD62L to define T cells that were phenotypically defined as naive (N), central memory (CM), effector memory (EM), or end-stage (ES) effector cells (Figures 2B,C). We also used a cocktail of nine antibodies to define subsets of NK, iNKT, and γ/δ T cells. In this case, the bioinformatic analysis included 13 distinct phenotypes (see Table S2 in Supplementary Material for full list). Figure S1 in Supplementary Material shows a representative plot of this panel and some of the subsets identified. In total, multi-color flow cytometry was initially performed on 23 patients using >150 samples collected at six- to eight-time points pre- and post-transplantation. An additional 5 patients were included in our study and independently analyzed to validate the prediction tool derived from the analysis of the first 23 patients.

We employed PCA as a powerful exploratory tool for revealing potential structure within the data set of the 23 patient cohort. The first few principal components often capture most of the information in the data and are therefore a very effective way to reduce the dimensionality of a high-dimensional data set. We initially based our analysis on the adaptive immune panel. Figure 2A shows a PCA based on T cell subset frequencies, as determined by our adaptive immune panel. Strikingly, patients who experienced rejection tend to cluster toward the left hand side, with PC1 scores <0. Conversely, patients who did not present with any signs of clinical rejection tend to have positive PC1 scores. Interestingly, the distinction between graft rejection and tolerance was lost when patient subset frequencies were normalized to pretransplant baseline measurements (Figure 2B). Differences between patients at baseline are therefore more important than relative differences post transplant in predicting graft prognosis post transplantation.

In contrast to the results obtained with the T cell panel, no clear stratification of rejection and tolerance was evident when the data obtained with the NK, iNKT, and γ/δ T immune panel were analyzed (Figure 2C). The frequency of the subsets identified by this panel therefore had no detectable predictive power for the development of rejection episodes in our cohort.

The first PCA component of the T cell panel suggested differential clustering of patients with and without rejection episodes. We therefore went on to investigate which T cell subsets were most important in driving this segregation. PCA allocates a “loading” to each dimension of the data (i.e., each T cell subset analyzed), which lie between −1 and 1. We identified those subsets with the largest, negative PC1 loadings (Table 2) to determine which subsets were implicated in predicting graft rejection. A common feature of all the largest loadings was the expression of PD1 in the identified T cell subsets. The largest negative loading in the CD8^+^ T cell population was seen in the PD1-expressing ES effector cells (CD45RO^−^/CD62L^−^). The negative loading in the CD4^+^ T cell population was similar in the N subset (CD45RO^−^/CD62L^+^), and in Treg cells (identified as CD25^+^ CD127 dull) with the ES (CD45RO^−^/CD62L^−^) phenotype.

**Table 2:** **The five largest negative loadings (predictive of rejection) obtained from PCA of T cell subset frequencies**.

T Cell subset	Loading
CD8^+^ CD45RO^−^ CD62L^−^ PD1^+^	−0.28
CD8^+^ PD1^+^	−0.25
CD4^+^ PD1^+^	−0.23
CD4^+^ CD25^+^ CD127^−^ CD45RO^−^ CD62L^−^ PD1^+^	−0.22
CD4^+^ CD45RO^−^ CD62L^+^ PD1^+^	−0.22

**Figure 3:**
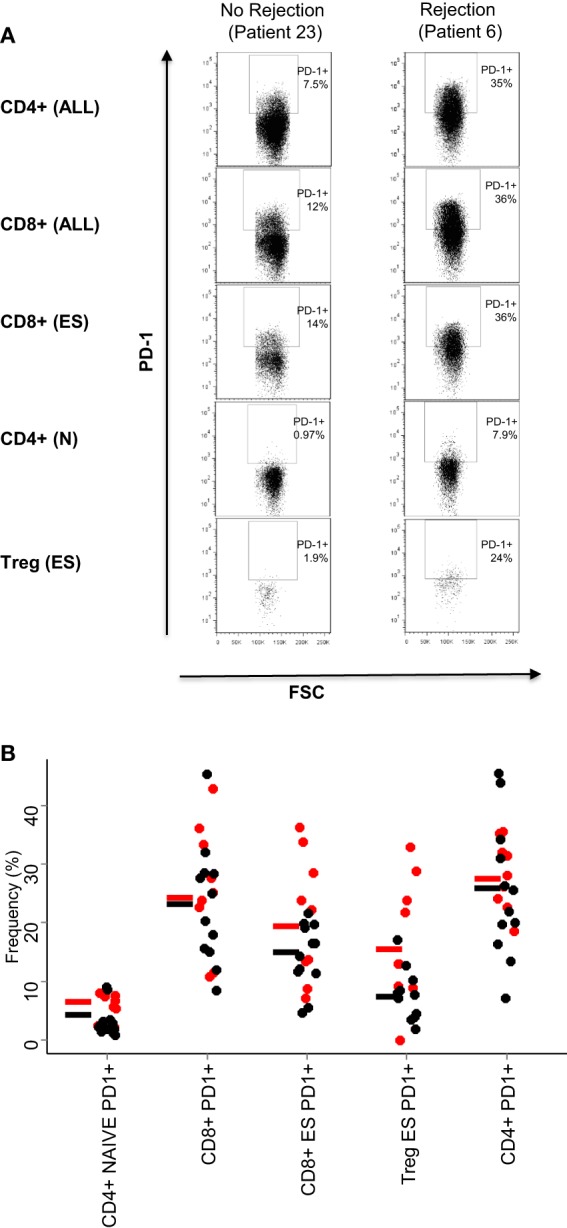
**PD1 expression on T cell subsets**. **(A)** Dot plots showing the expression of PD1 of the T cell subsets are indicated in Table 2: all CD4^+^ and CD8^+^ T cells, end-stage CD8^+^ T cells (CD8^+^, CD45RO^−^/CD62L^−^), naive CD4^+^ T cells (CD4^+^, CD45RO^−^/CD62L^+^), and end-stage Treg cells (CD4^+^ CD25^+^,CD127^−^, CD45RO^−^/CD62L^−^). **(B)** Relative frequencies (% of parent population) of the five T cell subsets with largest PCA loadings. The results for each patient are shown as a circle (red for rejectors, black for non-rejectors). No statistical differences were seen between the two patient groups for any markers shown (Mann–Whitney with Bonferroni correction).

On the basis of these results, we examined in more detail the expression of PD1 in the CD4^+^ and CD8^+^ T cell subpopulations in patients who had rejection episodes, compared to those who did not. Figure 3A shows flow cytometry plots of PD1 expression in a representative patient of each class. As predicted from the PCA analysis, the comparison of the flow cytometry plots showed that increased expression levels of PD1 was associated with rejection episodes. The summary of the PD1-expressing T cell subsets in all patient samples analyzed is shown in Figure 3B.

**Figure 4:**
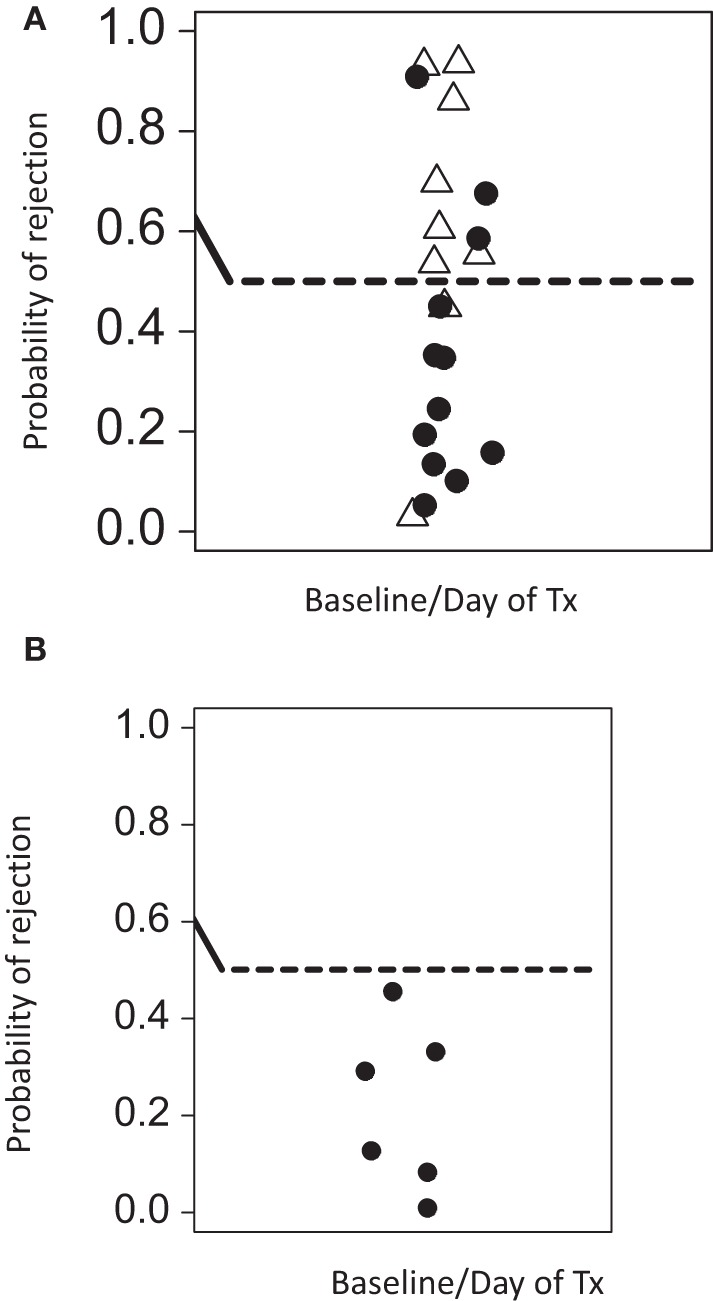
**Predicted rejection risk scores calculated from the pretransplant baseline SVM model**. **(A)** The SVM was constructed using only baseline data (at or immediately before transplantation) and risk scores for rejection [0,1] were derived from the model as described in Section “Materials and Methods.” A score of >0.5 is at risk of rejection. Open triangles are samples from patients with rejection episodes, and filled cycles from patients without rejection. **(B)** The SVM was consequently tested on a further cohort of five patients who were analyzed by flow cytometry by a scientist not involved in the initial studies. As control, one patient who was previously analyzed was re-tested in this independent analysis. None of these patients experienced rejection episodes. The SVM trained on the initial patients correctly predicts all patients in this validation analysis to be at low risk of rejection.

While PCA suggests that immune-phenotyping data can stratify patients with risk of transplant rejection, it is not designed to provide accurate predictions from new data. SVM are a class of very well-studied machine learning classification tools (see Materials and Methods). We constructed SVM classifiers based on data from three distinct time points during the course of renal transplantation: baseline, mid (between 4 and 6 weeks post-tx), and late (9–12 weeks post-tx). Using leave-one-out validation, we observed that baseline and midtime points correctly predicted the rejection status in 77 and 82% of the samples, respectively. The SVM based on later time points showed a poorer ability to discriminate, correctly predicting 68% of the samples. The SVM risk score (see Material and Methods) based on baseline/pretransplant phenotype alone (which was available for 20 patients, 9 rejectors, and 12 non-rejectors) is shown in Figure 4A. A SVM risk score of >0.5 suggests that the patient is more likely to exhibit a rejection episode, while a score of <0.5 suggests the patient is more likely not to show a rejection score. The further the risk score is from 0.5, the greater the confidence of the prediction.

We initially used leave-one-out validation because this provides the most powerful way to analyze the relatively small number of patients available for this study. However, we also used the more traditional train/test strategy. We selected the first (by date order of transplant) seven rejector and non-rejector data sets (in order to have a balanced data set) and used the data from these patients to build an SVM. We then used this SVM to predict the outcome of the remaining 10 patients. The SVM correctly predicted 7/10 of the remaining patients even using this small training set, providing further support for the robustness of the predictions. Finally, we used the SVM built on the first set of 23 patients analyzed to predict the rejection status of five further patients for whom baseline pretransplant samples were available and independently analyzed by a scientist who was not involved with the first cohort analysis. As a control for reproducibility, we included one sample of a patient of the first cohort in this independent analysis. All analyzed patients were correctly predicted to be non-rejectors (Figure 4B).

**Figure 5:**
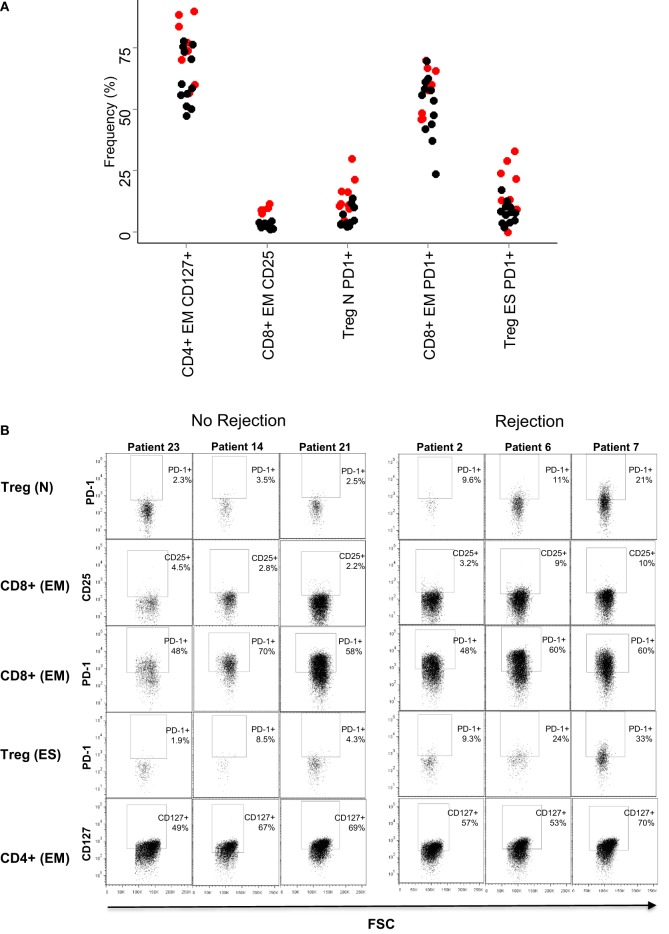
**Baseline frequencies and flow cytometry profiles of the subsets with largest coefficients in the SVM**. **(A)** Relative frequencies (% of parent population) of the five T cell subsets with largest SVM coefficients. The result for each patient is shown as a circle (red for rejectors, black for non-rejectors). Significance values (Mann–Whitney with Bonferroni correction) for the five subsets are: PD1^+^ naive Treg (CD4^+^ CD25^+^,CD127^−^, CD45RO^−^/CD62L^+^, PD1^+^) *p* = 0.02, CD25^+^ effector memory CD8^+^ T cells (CD8^+^, CD45RO^+^/CD62L^−^, CD25^+^) *p* > 0.1, PD1^+^ effector memory CD8^+^ T cells (CD8^+^, CD45RO^+^/CD62L^−^, PD1^+^) *p* > 0.1, PD1^+^ end-stage Treg (CD4^+^ CD25^+^,CD127^−^, CD45RO^−^/CD62L^−^, PD1^+^) *p* = 0.1, and CD127^+^ effector memory CD4^+^ T cells (CD4^+^, CD45RO^+^/CD62L^−^, CD127^+^) *p* > 0.1. **(B)** Dotplots showing PD1 (*y* axis) and FSC (*x* axis) expression in the five T cell subsets are described in **(A)**, in three representative patients that did not reject, and three representative patients that did reject.

The SVM generates a set of weights (between −1 and 1) equivalent to those generated by the PCA weights and corresponds to “loadings” given to each dimension of the data (i.e., each T cell subset analyzed) when generating the classifying hyperplane. We analyzed the weights given to each data subset by the optimized SVM. In agreement with the results of the PCA, three of the five subsets with the largest coefficients included PD1 (Table 3). The remaining two subsets consisted of CD8 and CD4 EM T cells expressing CD25 and CD127, respectively. The mean frequency (as a proportion of parent) of each of all five subsets is shown in Figure 5A, and flow cytometry plots of PD1 expression in three representative patients with and without rejection episodes are shown in Figure 5B.

**Table 3:** **The five largest positive loadings (predictive of rejection) obtained from SVM classification of T cell subset frequencies**.

T cell subset	Loading
CD4^+^ CD25^+^ CD127^−^ CD45RO^−^ CD62L^+^ PD1^+^	0.079
CD8^+^ CD45RO^+^ CD62L^−^ CD25^+^	0.076
CD8^+^ CD45RO^+^ CD62L^−^ PD1^+^	0.072
CD4^+^ CD25^+^ CD127^−^ CD45RO^−^ CD62L^−^ PD1^+^	0.066
CD4^+^ CD45RO^+^ CD62L^−^ CD127^+^	0.065

**Figure 6:**
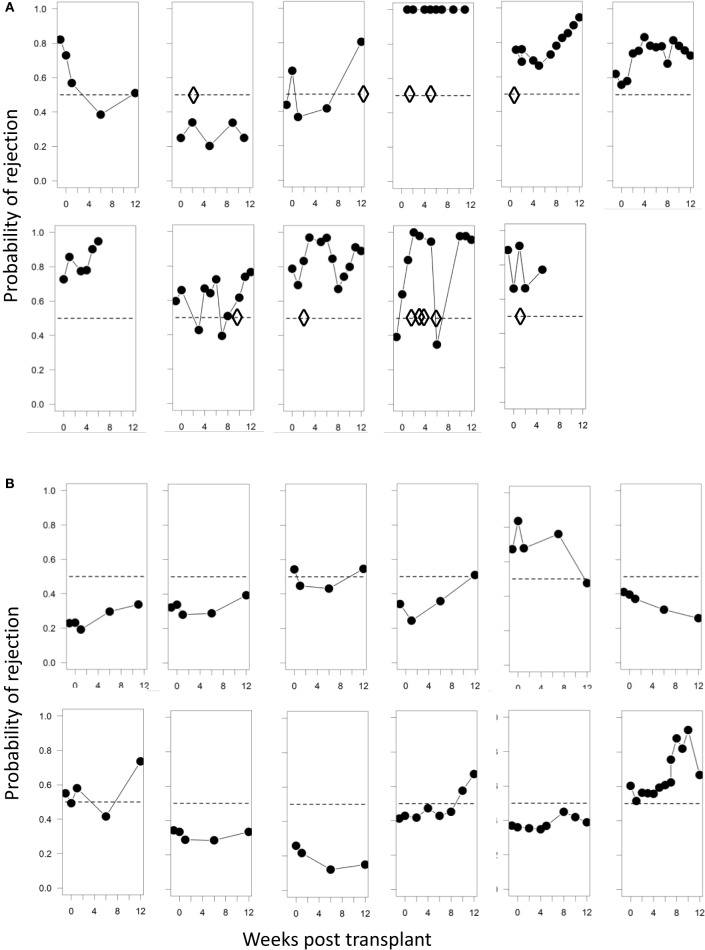
**Probabilities of identifying a rejection episode calculated from the SVM model**. **(A)** Trajectories of the probability of rejection determined by SVM for each patient who clinically presented with rejection. Diamonds represent each clinically observed rejection episode. In panel 1 rejection occurred at 6 months; in panel 6 at 12 months, and in panel 7 at 24 months. **(B)** Trajectories of the probability of rejection determined by SVM for patient number 12–23 (Table 1), who presented with no clinical signs of rejection. The SVM is trained solely on baseline samples and evaluated using leave-one-out cross-validation.

In addition to prediction of rejection prior to transplantation, it would be useful to use non-invasive screening to identify rejection post-transplantation without the need for biopsy. We therefore examined whether the immune-phenotype could identify rejection episodes using the SVM-derived probabilities of class membership (see Materials and Methods). We calculated the risk of rejection episodes over the 12-week period post-tx for each time point when a blood sample was collected and analyzed. Figure 6A shows the projected risk of rejection for each of the 11 patients who clinically presented with either cell- or antibody-mediated rejection; the time of rejection episodes is indicated with diamonds. In patients 1, 6, and 7, the rejection occurred after 12 weeks (see Table 1), which was outside the time period of collecting samples for this study. All but one of the remaining seven patients had a risk score >0.5, and five of the seven patients had a risk score greater than 0.75 at the time point when rejection episodes occurred. In contrast, only 4 out of 12 patients without rejection episodes had a risk score of more than 0.75 at any time point during the 12-week observation period (Figure 6B).

## Discussion

The major conclusion of our study is that the immune-phenotype of the peripheral blood T cell compartment contains information, which can predict the risk of a rejection episode following renal transplantation. In contrast, the panel defining NK, iNKT, and γ/δ T cells did not identify a phenotype, which segregated between rejection and non-rejection. This may reflect a dominant role of α/β T cells in acute graph rejection, but it is also possible that our “innate lymphoid” flow cytometry panel did not include markers that might identify subsets involved in regulating transplant tolerance or rejection. It should be noted that we have used a limited number of markers to identify the T cell subsets and the expression of activation and exhaustion markers. It is possible that revised panels that include additional markers might improve the ability to identify patients at risk of rejection. In this study, we have not explored the risk factors of antibody-mediated rejection, which is mediated by donor-specific antibodies that are present in patients before transplantation.

Advances in multi-color flow cytometry have led to a continual increase in the complexity of immune-phenotyping. The introduction of new technologies, such as mass-cytometry or single cell transcriptomics, has the potential to further increase the number of molecules that can be quantified (9, 10). However, the high cost and technical challenges of using these new technologies mean that flow cytometry remains the technique of choice for immune phenotyping in most clinical settings. In this study, we have used nine parameters, which generated a large number of possible combinations that cannot be comprehensively interrogated using manual approaches. In addition, there is an increasing awareness that a classification on the basis of a few defined phenotypes is a simplification of the underlying biology. Computational tools, which can efficiently mine the increasingly high-dimensional space of immune-phenotyping data, are therefore likely to become a key to future biomarker discovery and translation into the diagnostic laboratory.

We have used a hybrid approach, in which quantitative population frequency data is first collected using classical manual flow cytometry analysis and then analyzed using high-dimensional computational statistical tools. This combination of manual and computational analysis was used to define 52 different T cell subsets, many of which were nested in each other. The overall accuracy of the classification was >75% when using samples collected either before, at or within 6 weeks post transplant. These data provide a strong rationale for further immunophenotyping studies, with the dual objective of increasing the size of the training cohort and thus the power of the machine learning algorithm, and perhaps incorporating additional immunophenotyping markers, which might further dissect the intrinsic variation in the immune status of patients, and hence accurately predict the response to the transplant and the associated immunosuppression.

In addition to the ability to predict rejection episodes, two important further conclusions emerge from both the PCA and SVM analysis. The first is that the predictive power lies predominantly within the pretransplant immunophenotype, and that the phenotype predictive power becomes much weaker at later times. In the first few weeks after transplantation, there is remarkably little change in the immune phenotype, but as time progresses the phenotype of different patients seems to converge. This unexpected finding provides a rationale to potentially use the pretransplant immune phenotype to identify patients at risk of developing rejection episodes, and then increase immune suppressive medication during the early phase after transplantation.

The second finding is that high PD1 expression in several T cell subsets predicts a higher rate of rejection. The function of PD1 has been studied extensively (11), although its role in Treg is less well understood (12). PD1 up-regulation has been observed in murine models of chronic viral infection and was found to identify exhausted T cells with reduced function (13). Similar observations have been made in cancer patients, and the treatment with anti-PD1 antibodies has reversed T cell dysfunction and resulted in impressive clinical benefits for patients (14). Recent experiments have indicated that the PD1/PD-L1 pathway is involved in Treg-mediated suppression of autoreactive B cell responses (15). However, this did not involve PD1-positive Treg, as the suppression was mediated by PD-L1 expressed by Treg binding to PD1 expressed by the autoreactive B cells. Maybe more relevant for our observation is a recent study that demonstrated increased numbers of PD1-expressing Treg in the blood of patients suffering from an autoimmune condition that results in generalized vitiligo (16). Interestingly, upregulation of PD1 in Treg was also found in patients with chronic hepatitis C infection, and the observation that blockade of PD1 improved Treg function suggested that PD1 acted as negative regulator of Tregs in this setting (17). Together, these studies suggest that chronic immune activation (autoimmune or chronic infection) can result in the accumulation of PD1-expressing Treg with impaired functional activity. In our case, increased PD1 expression before transplantation might identify patients with a history of chronic immune activation combined with impaired Treg function, which together might enhance the potential of mounting damaging T cell responses against the transplanted kidney. This is in keeping with the recent demonstration that variations in the immune response profile of humans are strongly affected by environmental factors (18). It is possible that dialysis, age, and increased pathogen exposure might impact on the number of PD1-expressing Treg; in our cohort more patients were on dialysis and had a higher median age in the rejection group compared to the group without rejection. We note that an mRNA expression study in the peripheral blood of renal transplant patients showed that increased levels of PD1 mRNA was associated with acute rejection episodes (19). In this study, we have not analyzed the expression profile of CD57, a marker that has been linked to the resistance of renal transplant patients to respond to treatment with recombinant proteins that inhibit CD28 costimulation (20).

In conclusion, our study shows how computational tools, which are able to analyze the increasingly high-dimensional immunophenotyping data available, can be used to generate biomarkers useful for patient stratification, and to identify new biological features underlying a complex process such as transplant rejection. Validation of these results on a larger cohort is required, but this study suggests that immune-phenotyping may be useful in guiding patient management and may provide a strategy for developing personalized immune suppressive regimes according to the predicted rejection risk assessed prior to transplantation.

## Author Contributions

HS conceived and designed the work and wrote the paper. RP designed the work, acquired and analyzed data, and read and approved the paper. NT contributed to the design the work, analyzed data, read and approved the paper. SW contributed to the design the work, and read and approved the paper. LA contributed to the design the work and read and approved the paper. DG contributed to the design the work and read and approved the paper. SS acquired and analyzed data and read and approved the paper. MJ contributed to the design the work and read and approved the paper. MH, BC, and DT contributed to the design the work, analyzed data, and read and approved the paper. All authors agree to be accountable for all aspects of the work in ensuring that questions related to the accuracy or integrity of any part of the work are appropriately investigated and resolved.

## Conflict of Interest Statement

HS is consultant for Cell Medica and hold shares in this company. The remaining authors declare that the research was conducted in the absence of any commercial or financial relationships that could be construed as a potential conflict of interest.
