# Supplementary material for: PD1-Expressing T Cell Subsets Modify the Rejection Risk in Renal Transplant Patients
Source: Front Immunol. 2016 Apr 11;7:126. doi: 10.3389/fimmu.2016.00126 (PMC4827377; doi:10.3389/fimmu.2016.00126)
Supplement: Supplementary file 1 [file Table_1.DOCX]

**Table S1: T Cell Immune-Phenotyping Panel.** A table showing all subsets that were defined using the T cell flow-cytometric staining panel.

Supplementary Table S1
