# Supplementary figures and images for: PD1-Expressing T Cell Subsets Modify the Rejection Risk in Renal Transplant Patients
Source: Front Immunol. 2016 Apr 11;7:126. doi: 10.3389/fimmu.2016.00126 (PMC4827377; doi:10.3389/fimmu.2016.00126)

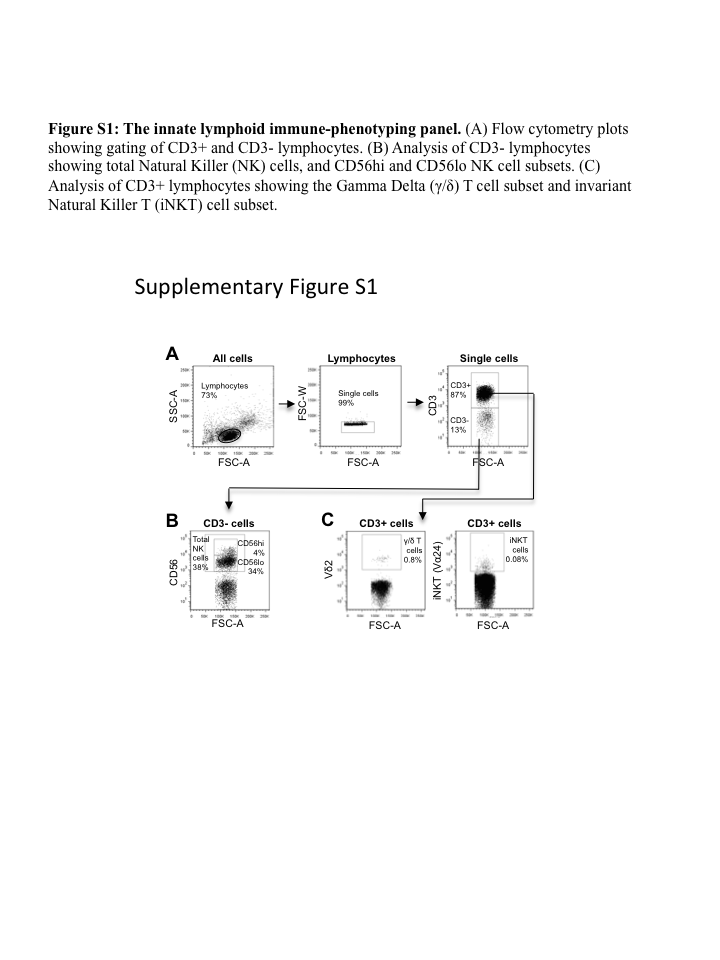

Supplement: Supplementary file 3 [file Image_1.TIFF]
